# Human papillomavirus infection in anal intraepithelial lesions from HIV infected Cuban men

**DOI:** 10.1186/s13027-017-0118-9

**Published:** 2017-01-17

**Authors:** Celia M. Limia, Yudira Soto, Yanara García, Orestes Blanco, Vivian Kourí, María V. López, María E. Toledo, Lissette Pérez, Yoanna Baños, Yaniris Caturla, Francisco Aguayo

**Affiliations:** 1 Institute of Tropical Medicine “Pedro Kourí”, Autopista Novia del Mediodía Km 6 ½, P.O.Box. 601, Marianao 13, La Habana Cuba; 2 General Hospital “Enrique Cabrera Cossío”, Calzada Aldabó 11117 esquina E, Boyeros, 10800 La Habana Cuba; 3 Virology Program, I.C.B.M., Faculty of Medicine, University of Chile, Independencia, 1027 Chile

**Keywords:** HPV, Anal squamous intraepithelial lesions, HIV, Anal cancer

## Abstract

**Background:**

An association between HPV infection and progression to anal squamous intraepithelial lesions (ASIL) has been established, specifically in high-risk populations such as HIV-infected men. In this population, anal cancer is one of the most common non-AIDS-defining malignancies.

**Methods:**

A cross-sectional study to detect anal lesions and HPV infection was performed. Anal mucosa samples were collected from 56 HIV-infected men from Cuba. The cytological diagnosis was done according to Bethesda 2001 System. HPV DNA detection was determined by qPCR for six high-risk HPV types and end point PCR for low-risk HPV types (6 and 11). The end point PCR with nucleotide sequencing technique was achieved to detect other genotypes of HPV not included in the qPCR in those samples negative for HPV- 6 and 11 or negative for the six genotypes identified in the qPCR.

**Results:**

Cytological diagnosis identified 53 of 56 (95%) men with abnormal anal cytology. Among those, 26% (14/53) had atypical squamous cells of undetermined significance (ASC-US), 4% (2/53) had atypical squamous cells of undetermined significance cannot exclude high-grade lesions (ASC-H), 64% (34/53) had low-grade squamous intraepithelial lesions (LSIL), and 6% (3/53) had high-grade squamous intraepithelial lesions (HSIL). HPV DNA was detected in 89% (50/56) of men and 79% had at least one of the high-risk HPV types. HPV- 16 was the most common genotype (52%), while HPV-18 was the most frequently detected genotype in men with HSIL. We found statistically significant differences in the HPV viral loads with respect to the cytology results (*p* = 0.0006) and that the practice of receptive anal sex was a risk factor for anal HPV infection (*p* = 0.032).

**Conclusion:**

This study shows a high prevalence of ASIL and high-risk HPV infections in the study group and is the first study showing the distribution of HPV genotypes in HIV infected Cuban men with abnormal anal cytology. This information may be of importance for local decision makers to improve prevention strategies, including the introduction of HPV vaccine in Cuba.

## Background

While anal carcinoma is rare in the general population [1], it is one of the most common non-AIDS-defining malignancies in the era of combination antiretroviral therapy [2]. It is more frequent among individuals with human immunodeficiency virus (HIV) infection, particularly in men who have sex with men (MSM) [3]. In the past decades, the incidence of anal cancer has been increasing in both general MSM and HIV-infected MSM [4]. The Cuban AIDS epidemic has a predominance of male HIV-infected individuals (81%), of which 89% are MSM [5].

Persistent human papillomavirus (HPV) infection, especially high-risk HPV (HR-HPV) viral types such as HPV- 16 and HPV- 18, is associated with anal cancer and its precursor lesions, high-grade anal squamous intraepithelial lesions (HSIL) [6]. Recent studies have demonstrated that more than 90% of HIV-positive MSM were also infected with HPV [2, 7]. This particular group has a very high risk of developing ASIL and anal cancer [8]. The development of anal squamous intraepithelial lesions (ASIL) is associated with HPV infection, as well as other risk factors such as receptive anal intercourse, history of sexually transmitted diseases, number of lifetime sexual partners, HIV status, lower CD4 cell count, and current cigarette smoking [9]. Screening of anal precancerous lesions in HIV-infected MSM has been suggested to be important for cancer prevention [10].

Little is known about anal HPV infection or anal cytological abnormalities in HIV-infected men in Cuba. The objective of this study was to investigate the distribution of HPV genotypes, and anal cytological abnormalities in HIV-infected men in Havana, Cuba.

## Methods

### Study design and population

We performed a cross-sectional study to detect ASIL and HPV infection. Study participants were outpatients of the sexually transmitted diseases (STD) clinic at the Institute of Tropical Medicine “Pedro Kourí” and were recruited between April and August 2012. Those eligible to participate were HIV-positive males with a history of a previous or current STD, willing to give informed consent to an interview and tests for anal HPV infection and anal cytology. The inclusion criteria also included having an anal cytology useful for diagnosis.

### Cytological analysis and HPV genotyping

Prior to the sample collection, the anal canal was cleaned carefully twice using a saline- moistened cotton-flocked swab. The cytology specimens were collected by mean of a pre-scored brush (QIAGEN, Hielden, Germany), placed on a glass slide, stained with a Papanicolaou stain and classified using the Bethesda system criteria for evaluation of cervical cytological results [11, 12].

After the cytology, another sample was collected for DNA extraction. The brush was then placed in a 1 mL Specimen Transport Medium^TM^ (QIAGEN, Hielden, Germany). DNA was obtained using the QIAmp DNA Mini kit (QIAGEN, Germany) following the manufacturer’s protocol.

HPV DNA detection was determined by real time polymerase chain reaction (qPCR) for six HR- HPV types (HPV −16, −18, −31, −33, −45 and −58), as described below. HPV- 6 and 11 were detected using type specific end point PCR with L1 primers [13]. The end point PCR with nucleotide sequencing technique was achieved to detect other genotypes of HPV not included in the qPCR in those samples negative for HPV- 6 and 11 or negative for the six genotypes identified in the qPCR.

### Real time polymerase chain reaction

The methodology used in this study is based on a protocol previously published by Schmitz et al. in 2009 [14] and it has already been described in detail in a previous article by our group [15].

PCR primers and corresponding TaqMan probes were used for HPV types −16, −18, −31, −33, −45, and −58 for target amplification of the LCR/E6/E7 regions of HPV genome. To identify and quantify viral load of those HR-HPV types, single PCR reactions were performed for each HPV type. To control for DNA quality, β-globin was amplified for each sample, in one of the reactions. Sequences of primers and probes are listed below (Table 1).

**Table 1:** Primer and probes for real time polymerase chain reaction

LCR/E6/E7 regions primers and TaqMan probes	Sequences 5′- 3′
HPV 16 +	5′ gaa ccg aaa ccg gtt agt ata a 3′
HPV 16 -	5′ atg tat agt tgt ttg cag ctc tgt 3′
HPV 16 Probe	5′ cat ttt atg cac caa aag aga act gca atg ttt c 3′
HPV 18 +	5′ gga ccg aaa acg gtg tat ata a 3′
HPV 18 -	5′ cag tga agt gtt cag ttc ggt 3′
HPV 18 Probe	5′ atg tga gaa aca cac cac aat act atg gcg cg 3′
HPV 31 +	5′ gaa ccg aaa acg gtt ggt ata ta 3′
HPV 31 -	5′ atc gta ggg tat ttc caa tgc 3′
HPV 31 Probe	5′ cat agt att ttg tgc aaa cct aca gac gcc atg t 3′
HPV 33 +	5′ gca tga ttt gtg cca agc at 3′
HPV 33 -	5′ ctc aga tcg ttg caa agg ttt 3′
HPV 33 Probe	5′ act ata cac aac att gaa cta cag tgc gtg gaa tgc 3′
HPV 45 +	5′ cag tgt aat aca tgt tgt gac cag 3′
HPV 45 -	5′ aca gga tct aat tca ttc tga ggt 3′
HPV 45 Probe	5′ caa gaa aga ctt cgc aga cgt agg gaa aca c 3′
HPV 58 +	5′ cac gga cat tgc atg att tgt 3′
HPV 58 -	5′ tca gat cgc tgc aaa gtc ttt 3′
HPV 58 Probe	5′ ttt caa ttc gat ttc atg cac 3′
β globin +	5′ aca caa ctg tgt tca cta gc 3′
β globin -	5′ caa ctt cat cca cgt tca cc 3′
β globin Probe	5′ tca aac aga cac cat ggt gca tct gac tcc 3′

HPV-16 and 18 standard curves were obtained from purified genomic DNA of SiHa and HeLa cell lines, respectively. HPV16-positive cervical carcinoma cell line SiHa (2 genome copies of HPV-16) and the HPV18-positive cell line HeLa (10–50 genome copies of HPV-18) were used to generate standard DNA for measurements of viral load (tenfold genome dilutions between 10^6^ and 10 copies/μL).

The pattern curves were constructed on the basis of each of the resulting standard DNA, which showed good linear correlation (r: 0, 99) and low error values throughout 6 target DNA concentrations. The system had a lower detection limit of 10 copies for HPV-16 and other genetically related genotypes such as HPV- 31, −33 and −58. The lower detection limit for HPV-18 and other genetically related genotype, HPV-45, was also of 10 copies of viral genome. No cross reactions between HPV types and other DNA viruses were observed [14, 16].

PCR was performed in a Light Cycler 1.5 platform (Roche Molecular Biochemical of Indianapolis, USA). The PCR reaction comprises 5 μL of DNA (up to 50 ng), 4 μL of Quantitative PCR TaqMan Master Mix (Roche Molecular Biochemical of Indianapolis, USA), 10 pmol of each primer and 1 to 5 pmol of each probe in a final volume of 25 μL. The initial denaturation step at 94 °C for 10 min was followed by 45 PCR cycles at 94 °C for 15 s, 50 °C for 20 s and 60 °C for 40 s each.

HPV viral load was measured using absolute quantification. The viral load values were expressed as copies/μL. Cases with more than one HPV type were reported as multiple infections.

### End point PCR and sequencing

The amplification reactions included a set of degenerated primers MY09/MY11 [17]. Each amplification reaction was performed in a total volume of 50 μL. The reaction mixture contained nuclease-free water, 10 mM Tris–HCl (pH 8.3), 50 mM KCl, 6 mM MgCl^2^, 200 μM of each dNTP, 5 μM of MY primers, 2,5-U Taq Polymerase (Roche, Diagnostics, Indianapolis, USA), and 100 ng of each DNA. Each PCR was performed with first denaturation step at 95 °C for 10 min, followed by 45 PCR cycles at 95 °C for 1 min, 55 °C for 1 min and 72 °C for 1 min. A final extension at 72 °C for 10 min was included.

Polymerase chain reaction products of positive samples were purified using QIAquick® PCR purification kit (QIAGEN, Hilden, Alemania). These were subsequently sequenced using 1 μL of 5 μM of MY09 and MY11 as the sequencing primers, 8 μL of sequence reaction mixture DTCS Quick Star Master Mix supplied with the Dye Terminator Cycle Sequencing (DTCS) Quick Start Kit (Beckman Coulter, Fullerton, CA), 5 μL of purified DNA and 6 μL of water to complete 20 μL of reaction mixture. The sequencing reaction consisted of 2 min denaturation at 96 °C, followed by 50 cycles of denaturing at 96 °C for 20 s, 20 s of hybridization at 50 °C, and 4 min of extension at 60 °C. Once the sequence reaction was concluded, it was purified, after the protocol described in the DTCS Quick Star Master Mix commercial kit (Beckman Coulter). The sequencing fragments were run on the genetic analysis system CEQ 8800 (Beckman Coulter). Finally, the sequences were edited and assembled using Sequencher, version 4.10 (Gene Codes Corporation, Ann Arbor, MI). Human papillomavirus genotype was determined using Basic Local Alignment Search Tool (BLAST, www//ncbi.nih.gov) and confirmed by manual phylogenetic analysis, using CLUSTAL-X and the neighbor-joining method in MEGA version 5 (Kimura’s 2-parameter correction, bootstrap 1000) [18].

### Statistical analysis

Data were processed using IBM SPSS Statistics 19. Odds ratios (ORs) and 95% confidence intervals (CIs) were calculated, using univariate logistic regression, as estimates of the association of HPV infection and anal cytology results with potential risk factors. Comparisons between viral loads from groups of patients with different cytological classifications were made using the Kruskall-Wallis and Mann—Whitney tests. All statistical tests were considered to be significant at a *p* value of < .05.

## Results

A total of 56 eligible participants consented to be included into this study. The socio-demographic, epidemiological, and clinical characteristics of the participants are shown in Table 2. The median age of the participants was 35 years (range, 20–61 years). The majority of individuals (89%) described themselves as MSM, and 66% of these had their first intercourse at 15 years old or later. Around 84% of the participants have STD other than HIV at the time of this study, and 44 (79%) were diagnosed for anogenital condyloma. Thirty (54%) participants had a recent CD4 cell count between 200 to 500 cells/mL of blood, and 79% participants ever had highly active antiretroviral therapy (HAART). About 34% of the participants reported ≥5 anal sex partners during the last two years.

**Table 2:** Relationship between anal HPV infection and socio-demographic, epidemiologic and clinical risk factors in the studied population

Variables		*N* (%)	HPV-positive *N* (%)	HPV-negative *N* (%)	*p* value	OR (CI 95%)
Total		56 (100)	50 (89)	6 (11)		
Age, median (IQR)		35 (27–46)	_	_		
Age (years)					0.862	
	20–29	22 (39)	20 (40)	2 (33)		2.50 (0.30–20.92)
	30–39	11 (20)	11 (22)	0 (0)		---
	40–49	13 (23)	11 (22)	2 (33)		1.38 (0.16–11.94)
	≥50	10 (18)	8 (16)	2 (33)		1 (Reference)
Education					0.766	
	Primary and junior high school	15 (27)	14 (28)	1 (17)		2.55 (0.20–31.86)
	High School	28 (50)	25 (50)	3 (50)		1.52 (0.22–10.38)
	University	13 (23)	11 (22)	2 (33)		1 (Reference)
Marriage status					0.999	
	Married	9 (16)	8 (16)	1 (17)		1 (Reference)
	Unmarried	43 (77)	38 (76)	5 (83)		0.95 (0.10–9.27)
	Divorced	4 (7)	4 (8)	0 (0)		---
Self-reported sexual orientation					0.084	
	MSM	50 (89)	46 (92)	4 (67)		5.75 (0.79–41.69)
	Heterosexual	6 (11)	4 (8)	2 (33)		1 (Reference)
Alcohol consumption					0.196	
	Yes	23 (41)	19 (38)	4 (67)		0.31 (0.05–1.84)
	No	33 (59)	31(62)	2 (33)		1 (Reference)
Smoking					0.229	
	Yes	24 (43)	20 (40)	4 (67)		0.33 (0.6–2.00)
	No	32 (57)	30 (60)	2 (33)		1 (Reference)
CD4+ T cells count					0.465	
	<200	17 (30)	16 (32)	1 (17)		4.57 (0.35–59.11)
	200–500	30 (54)	27 (54)	3 (50)		2.57 (0.36–18.49)
	>500	9 (16)	7 (14)	2 (33)		1 (Reference)
Age of first sexual intercourse (years)					0.362	
	<15	19 (34)	18 (36)	1 (17)		2.81 (0.30–25.98)
	≥15	37 (66)	32 (64)	5 (83)		1 (Reference)
Sexual practice					0.492	
	Genital	5 (9)	4 (8)	1 (17)		1 (Reference)
	Oral/Genital	51 (91)	46 (92)	5 (83)		2.30 (0.21–24.80)
Anal receptive sex					*0.032*	
	Yes	47 (84)	45 (90)	3 (50)		*9.00 (2.42–57.12)*
	No	9 (16)	5 (10)	3 (50)		1 (Reference)
Number of sexual partners during the last two years.					0.756	
	1	19 (34)	16 (32)	3 (50)		1 (Reference)
	2–4	18 (32)	17 (34)	1 (17)		3.19 (0.30–33.89)
	5–10	8 (14)	8 (16)	0		---
	>10	11 (20)	9 (18)	2 (33)		0.84 (0.12–6.03)
HAART					0.459	
	Yes	44 (79)	40 (80)	4 (67)		1 (Reference)
	No	12 (21)	10 (20)	2 (33)		0.50 (0.08–3.13)
STD at the time of the study (except HIV)					*0.031*	
	Yes	47 (84)	44 (88)	3 (50)		*7.33 (1.20–44.96)*
	No	9 (16)	6 (12)	3 (50)		1 (Reference)
History of STD (except HIV)					0.690	
	Yes	43 (77)	38 (76)	5 (83)		0.63 (0.07–5.97)
	No	13 (23)	12 (24)	1 (17)		1 (Reference)

The values with statistical significance appear in italic letters

*HPV* Human papillomavirus, *OR* Odd ratio, *IQR* Inter-quartile ratio, *CI* Confidence interval, *MSM* Men who have sex with men *STD* Sexually transmitted diseases, *HIV* Human immunodeficiency virus, *HAART* High activity anti-retroviral therapy

Overall, HPV DNA was detected in 89% (50/56) men and 79% (44/56) had at least one of the six HR-HPV types that we sought. The most common types detected were HPV-16 (52%), HPV-11 (48%), HPV-6 (41%) and HPV-18 (30%). Infection with multiple HPV types was detected in 71% of the participants (40/56), (Table 3). We also fund two cases positive for HPV- 53 (possibly carcinogenic genotype) and HPV-83 (genotype of unknown carcinogenicity), through generic PCR and DNA sequencing.

**Table 3:** HPV types distribution in HIV positive men according to anal cytology classification

HPV type	Cytology classification					
	NLIM *N* = 3 *n* (%)	ASC-US *N* = 14 *n* (%)	LSIL *N* = 34 *n* (%)	ASC-H *N* = 2 *n* (%)	HSIL *N* = 3 *n* (%)	Total *N* = 56 *n* (%)
HR-HPV types	3 (100)	9 (64)	27 (79)	2 (100)	3 (100)	44 (79)
HPV-16	2 (67)	6 (43)	17 (50)	2 (100)	2 (67)	29 (52)
HPV-18	1 (33)	1 (7)	11 (32)	1 (50)	3 (100)	17 (30)
HPV-31	0	1 (7)	5 (15)	0	1 (33)	7 (12)
HPV-33	1 (33)	3 (21)	11 (32)	0	0	15 (27)
HPV-45	1 (33)	2 (14)	5 (15)	1 (50)	0	9 (16)
HPV-58	2 (67)	0	8 (24)	0	2 (67)	12 (21)
HPV 16–18 only	1 (33)	0	4 (12)	0	1 (33)	6 (11)
HPV 16–18 and other HR-HPV	1 (33)	0	7 (21)	1 (50)	2 (67)	11 (20)
Multiple infections	3 (100)	7 (50)	26 (76)	1 (50)	3 (100)	40 (71)

HR-HPV types (16, 18, 31, 33, 45 and 58)

Concerning cytological findings, 53 of 56 (95%) men had abnormal anal cytology. Among those with abnormal cytology, 26% (14/53) had ASC-US, 4% (2/53) had atypical ASC-H, 64% (34/53) had LSIL and 6% (3/53) HSIL.

HPV prevalence was remarkable high in both normal and abnormal anal cytology. All cases with normal anal cytology had HPV infection (3/3). Among those with abnormal anal cytology, 89% (47/53) were positive for at least one of HPV type and 74% (39/53) were infected with multiple HPV types. Among the HPV-positive cases with abnormal anal cytology, 87% (41/47) had at least one HR-HPV genotype and 55% (26/47) had more than two HR-HPV types. The most prevalent HR-HPV types among cases with abnormal cytology were HPV-16 (51%, 27/53) and HPV-18 (28%, 15/53). HPV-16 was the major genotype in men with ASC-US, LSIL or ASC-H, followed by HPV- 33 in men with ASC-US, HPV-18 and 33 in men with LSIL and HPV-18 and 45 in ASC-H. HPV-18 was the most frequently detected genotype in men with HSIL, followed by HPV-16 and 58. In men with normal cytology, HPV-16 and 58 were the most prevalent types detected, (Table 3).

**Fig. 1:**
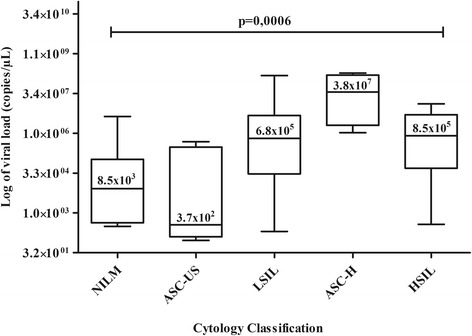
Comparison of the medians of HPV viral load among each anal cytology classification. Abbreviations: ASC-US: atypical squamous cells of undetermined significance, ASC-H: atypical squamous cells of undetermined significance cannot exclude high-grade lesions, LSIL: low-grade squamous intraepithelial lesions, HSIL: high-grade squamous intraepithelial lesions, NILM: negative for intraepithelial lesion and malignancy

Viral load analysis was detected for different anal cytological classifications. HPV viral load levels were significantly different among those with different anal cytology results (*p* = 0.0006). Those classified as negative and ASC-US showed lower viral load (median: 8.5×10^3^ copy/μL, ranges 3.2x10^2^ to 4.6x10^6^ copy/μL and median: 3.7×10^2^ copy/μL, ranges 9.4×10^1^ to 5.1×10^5^ copy/μL, respectively). In contrast, higher viral load was detected in cases with LSIL, ASC-H and HSIL (median: 6.8×10^5^ copy/μL, ranges 2.1×10^2^ to 1.6×10^8^ copy/μL, median: 3.8×10^7^ copy/μL, ranges 1.1×10^6^ to 2.0×10^8^ copy/μL and median: 8.5×10^5^ copy/μL, ranges 3.8×10^2^ to 1.4×10^7^ copy/μL, respectively) (Fig. 1).

Table 2 shows the association between HPV infection and socio-demographic, epidemiological, and clinical variables analyzed in the studied population. The participants who had receptive anal sex as a regular sexual behavior were at higher risk to HPV infection (*p* = 0.032; OR = 9.00 CI 95% 2.42-57.12). Sexually transmitted diseases were also a risk factor for viral infection (*p* = 0.031; OR = 7.33 CI 95% 1.20-44.96). Regarding HPV infection, according to age distribution, viral infection was not associated with age but a slight pick in prevalence for the 20- to 29-years old individuals was observed.

The population with ASIL/ASC-H was compared with those with normal anal cytology and ASC-US. A higher prevalence of ASIL/ASC-H was observed among those having oral/genital sex as a regular sexual behavior (*p* = 0.026; OR = 11.69 CI 95% 1.20-114.31). In addition, a positive diagnosis of STD, predominantly anal condyloma, was found to be related with an increased risk of ASIL/ASC-H (*p* = 0.017; OR = 6.55 CI 95% 1.40-30.58).

## Discussion

In the past decade, multiple studies have reported that anal HPV infection is common among HIV infected men [19–21]. In Cuba, there are few data about HPV prevalence in HIV-infected men [22]. In the present study a very high prevalence of HPV infection was found in both, individuals with abnormal and normal anal cytology. This high prevalence had been also found in various studies developed in HIV-infected men [7, 8, 23]. Torres et al. found a global HPV prevalence of 95.8% (95% CI, 94.6 to 96.7). They also found that patients with abnormal cytology presented a higher prevalence of any HPV type (99.7%), than those with negative cytology results. However, patients with negative results also had a very high prevalence of any HPV type (94.1%) [7]. Sahasrabuddhe et al. detected that the prevalence of any HPV genotype was 94.4% (95% CI, 91.4%–96.6%), and the prevalence of any carcinogenic HPV genotype was 75.4% (95% CI, 70.5%–79.9%). They found anal HPV infection in 98.4% of patients with HSIL and in 89.2% of men with nondysplastic biopsy findings [8]. These high prevalence rates could be explained by the effect of chronic HIV infection on normal immune function, which increases susceptibility to HPV infection and retention [24]. The mechanisms that facilitate this are still unknown.

The most common types detected were HPV-16, HPV-11, HPV-6, and HPV-18, which are included in the currently used tetravalent HPV vaccine. HR-HPV types were frequently detected for both, individuals with abnormal and normal anal cytology. These results are similar to studies from different geographic areas [7, 23]. However, Colon-Lopez et al. detected mostly HPV-58, 31 and 51 in high-risk men attending a sexually transmitted infection clinic in Puerto Rico [25]. The different prevalence and distribution of anal HPV subtypes in different study populations is of interest since it might reflect different geographic distributions of HPV genotypes or heterogeneous host susceptibility to the pathogens.

Our cytology results show that the prevalence of abnormal anal cytology overall was remarkably high, primarily with LSIL. Only 5.4% (3/56) of the participants had normal anal cytology.

It is difficult to directly compare the results from different studies due to the various characteristics of the study participants including age, stage of disease, status of immune system, and history of HAART. Salit and colleges, in 2010, evaluated the prevalence of abnormal anal cytology based on a cross-sectional study of 401 HIV-positive MSM from Canada. They found the highest prevalence of LSIL (43%). In a study of 247 HIV-Infected MSM, Pokomandy et al. found a prevalence of abnormal anal cytology of 80.9% [26]. In the present study, the prevalence of abnormal anal cytology was higher. According to these figures, further investigations are needed to confirm the high prevalence of cytological anal abnormalities in Cuban HIV positive men.

Our results showed receptive anal sex and other STD, specifically anal warts as risk factors for HPV infection in the studied population. A past history of receptive anal intercourse and recent receptive anal intercourses are the most consistently reported risk factors for prevalent anal HPV infections [27]. A recent history of anal warts and others STD have been described as important risk factors associated with the prevalence of anal HPV infections and anal cancer precursor lesions [21, 28].

Among MSM, there are few data on age-specific HPV prevalence. In our study HPV detection was very high and viral infection was not associated with age. However, we observed a slightly high prevalence of anal HPV in men aged 20- to 29- years.

The majority of studies found a high prevalence of anal HPV that did not decline with age [29]. Contrary, in a study by Gao et al. of 578 MSM (528 HIV-negative and 50 HIV-positive) recruited from two cities in China, a decrease in anal HPV prevalence was observed with increasing age (71% for ≤ 19 years, 62% for 20–29 years, 64% for 30–39 years and 54% for ≥ 40 years); however, this was not statistically significant [30]. In another study by Nyitray et al. of 179 HIV-negative MSM recruited through advertisements and from a genitourinary clinic in three sites in Brazil, Mexico and the US, a significantly decreasing age-specific prevalence of anal HPV was observed, with the highest HPV rates reported in the 18- to 24-year-old age group. These age-specific HPV infection prevalence trends may result from age-specific sexual behavior [31]. Difference source populations may explain the different age-specific estimates [29].

The significance of the high incidence of HPV detection in this population is uncertain. One potential explanation for the high prevalence of anal HPV infection among MSM and the lack of association with age is that HPV infection in the anus is more persistent than in the cervix. However, the persistence of some HPV types may not reflect the true persistence but rather clearance of an HPV type followed by subsequent exposure or reinfection with the same type [29].

In our study, higher levels of HPV viral load were associated with abnormal anal cytology. Tamalet et al. reported that HPV 16 viral load was an independent factor for abnormal anal cytology [32]. Rodel and colleagues found that HPV 16 DNA load and p16 (INK4a) expression were significant prognostic factors for local tumor control and overall survival of patients with anal cancer following chemo-radiotherapy [33]. However, Poizot-Martin et al. found that there was not association between HPV 16/18 viral loads and abnormal anoscopic results [34]. Longitudinal studies are needed to evaluate the link between high anal HPV DNA load and progression to anal squamous intraepithelial lesions and anal cancer.

Some limitations of this study should be considered. The small sample size of this study limited the analysis when interpreting the prevalence data, mainly to perform statistical association analysis. On the other hand, even when anal cytology results were confirmed by biopsy using standard anoscopy and patients were treated properly, this study had no access to biopsy results and to the outcome of each patient, because they were attended at coloproctology consults from different hospitals.

## Conclusion

In this study we found for the first time in Cuba, a high prevalence of HR-HPV infection in anal mucosa of HIV infected men. Because a large number of cases have shown cytological abnormality, we suggest the possibility of including a screening for HPV detection and genotyping in anal cancer precursor lesions of HIV seropositive individuals. This would facilitate the clinical management of these patients in the presence of anal cancer precursor lesions, taking into account the increased incidence of this disease in this risk group. Moreover, the information reported here may be useful to consider the possibility of including HPV vaccination in Cuba.

## Abbreviations

ASC-H: Atypical squamous cells of undetermined significance cannot exclude high-grade lesions
ASC-US: Atypical squamous cells of undetermined significance
ASIL: Anal squamous intraepithelial lesions
CIs: Confidence intervals
HAART: Highly active antiretroviral therapy
HIV: Human immunodeficiency virus
HPV: Human papillomavirus
HR-HPV: High risk-human papillomavirus
HSIL: Had high-grade squamous intraepithelial lesions
LSIL: Low-grade squamous intraepithelial lesions
MSM: Men who have sex with men
ORs: Odds ratios
qPCR: Quantitative real time polymerase chain reaction
STD: Sexually transmitted disease
